# Recommendations for the Use of Leeches in Reconstructive Plastic Surgery

**DOI:** 10.1155/2014/205929

**Published:** 2014-02-06

**Authors:** Kosta Y. Mumcuoglu

**Affiliations:** Parasitology Unit, Department of Microbiology and Molecular Genetics, The Kuvin Center for the Study of Infectious and Tropical Diseases, The Hebrew University, Hadassah Medical School, P.O. Box 12272, Jerusalem 91120, Israel

## Abstract

A written informed consent should be obtained from the patient before hirudotherapy is initiated. The patients should be treated each day of leech therapy with anti-*Aeromonas* antibiotics. Leeches should be applied on the darker spots of the reattached body parts or flaps. Usually 1–10 leeches are used for each treatment, while at the beginning, the patient might need two or more treatments per day. Leech therapy is used until venous capillary return is established across the wound border by angiogenesis. Usually the treatment with leeches lasts for 2–6 days. Hematologic evaluations should be performed every 4 hrs and the patient has to receive blood transfusions when the hemoglobin level is lower than 8 g/dL. Signs of regional lymphadenitis, slight swelling, and pain of regional lymph nodes on the side of leech application and subfebrile temperature can occur. Contraindications related to hirudotherapy include arterial insufficiency, hemophilia, hemorrhagic diathesis, hematological malignancies, anemia, hypotension, and sepsis. Leech therapy is not recommended in pregnancy and lactation and in patients with an unstable medical status, history of allergy to leeches or severe allergic diathesis, and disposition to keloid scar formation, as well as in those using anticoagulants and immunosuppressants.

## 1. Introduction

Medicinal leeches have been used in the past 50 years for the salvage of tissue with venous congestion. In 1960, Deganc and Zdravic [1] conducted the first treatment of congested flaps using leeches. Today, especially in the field of reconstructive microsurgery, medicinal leech therapy is enjoying a renaissance (for review see also [2, 3]).

Leeches are generally used during the critical postoperative period when venous outflow cannot match the arterial inflow, which can lead to venous congestion, clinically identified by the dusky purple appearance of the skin. If this complication is not corrected, cell death may result and the flap or finger may be lost. Therefore, medicinal leeches are used to salvage compromised microvascular free-tissue transfers, replanted digits, ears, lips, and nasal tips until angiogenesis gradually improves the physiological venous drainage [4]. Frodel et al. [5] used medicinal leeches to salvage soft tissue avulsion in key facial structures of 4 patients involving avulsions of the ear, nose, lip, and scalp. In addition to using leech therapy in head and neck reconstruction, there are numerous studies showing the use of leech therapy for hematomas, penile and total scalp replantation, and pedicled skin flaps, as well as for the salvage of the entire lower limp [6–19].

Leech therapy is usually initiated after failure of more conventional treatment modalities such as warming, aspirin, rheomacrodex (i.v.), immobilization and elevation of the injured area, and use of local heparin and vasodilators to improve venous status. Venous obstruction causes microcirculatory thrombosis, platelet trapping, and stasis. Thus, even after successful reanastomosis, secondary changes in the microcirculation can persist and prevent adequate outflow from being reestablished. Free flaps, pedicled flaps, and replanted tissues can survive arterial insufficiency for up to 13 hours, but venous congestion can cause necrosis within three hours. Medicinal leeches may be helpful in treating tissues with venous insufficiency by establishing temporary venous outflow, until graft neovascularization takes place [20].

In July 2004, the FDA approved leeches as a medical device in the field of plastic and reconstructive surgery. A survey of all 62 plastic surgery units in the United Kingdom and the Republic of Ireland showed that the majority of these units uses leeches postoperatively [21].

The aim of these recommendations is to review the practical use of medicinal leeches in reconstructive plastic surgery by reporting our experience with leeches in cases of reimplanted digits and free-tissue transfers.

## 2. Conditions for Treatment with Leeches

Once venous congestion has been identified and the patient has agreed to undergo leech therapy, it is important that the patient is informed about the benefits and potential risks of the treatment. A written informed consent should be obtained from the patients, their parents, and/or the legal guardian before hirudotherapy is initiated.

There is a general consensus that antibiotic prophylaxis for the *Aeromonas* bacteria, which are symbionts of leeches and which could lead to complications, should be initiated before leech therapy [22, 23]. *Aeromonas* species are sensitive to second- and third-generation cephalosporins, fluoroquinolones, sulfamethoxazole-trimethoprim, tetracycline, and aminoglycosides, while *Aeromonas* is resistant to penicillin, ampicillin, first-generation cephalosporins, and erythromycin [16, 24–27]. Patients should be treated each day of leech therapy with anti-*Aeromonas* antibiotics such as 500 mg of ciprofloxacin [22]. However, out of 21 isolates of *Aeromonas* species isolated from the water collected from the leech tanks, 71.4% were ciprofloxacin susceptible. All isolates were sulfamethoxazole-trimethoprim susceptible, which was also used as a prophylactic antibiotic regimen of choice for leech therapy [28]. A regular surveillance to detect resistant *Aeromonas* species in medical leeches, by controlling the water in which they are kept, was suggested. Chepeha et al. [29] used an antibiotic prophylaxis with double coverage during leech application and single coverage for 2 weeks after leech therapy is discontinued. In the Iowa Head and Neck Protocol [30], levaquin is administered before the first leech is applied to the skin and continued until 24 hours after leech therapy is discontinued.

Use of narcotics and benzodiazepines should be minimized, because they could negatively influence leech activity, while the treated area should be photographed under the same light conditions using the same camera periodically to follow the progress of decongestion [29].

## 3. The Medicinal Leech

According to the literature, in most cases the medicinal leech *Hirudo medicinalis *was used. In some cases, similar results were also obtained with *Hirudo verbana *and *Hirudo michaelseni* [17, 31]. Using mitochondrial sequences and nuclear microsatellites, Siddall et al. [32] demonstrated that there are at least three species of European medicinal leech and that many of the leeches marketed as *H. medicinalis* in the USA, France, and Germany are actually *H. verbana*.

Leeches should be purchased from recognized leech producing companies, where they are kept in appropriate farms and fed artificially with animal blood. In addition, leeches purchased from recognized leech farms are sufficiently starved prior to being sold and some of the leech farms have been approved by regulatory agencies. Leeches, which were collected from a natural environment, should not be used for hirudotherapy.

Leeches can survive one year or more without a blood meal. Therefore, it is recommended to keep a large number of leeches in the laboratory by changing the water once or twice weekly. For this purpose, the leeches can be transferred every week to a second container, which was previously cleaned and rinsed thoroughly to remove any remains of disinfectants and where the water was kept for at least 24 hrs for dechlorination. The container should be filled up to 3/4 with water and covered with a towel or netting, which would allow the leeches to have access to fresh air, without being able to escape. A larger stone should be added into the container, which would help leeches during the process of shedding their integument. The container with leeches should be kept in a cool place (preferably 4–15°C). When leeches are kept at room temperature, attention should be paid that the ambient temperature does not exceed 25°C and that the container with leeches is not exposed to direct sunlight. When replacing leeches from one container to the other, the replacement water must be at the same temperature as the original. Approximately 8 leeches should be kept in 1 liter of water.

Leeches should be handled gently by wearing nitrile or latex gloves, which would also prevent leeches from biting the health provider during maintenance of or treatment with leeches.

## 4. Application of Leeches to the Skin

Before application, leeches are thoroughly rinsed with deionized water. The area to be exposed to leeches should be cleaned with sterile distilled water and ointments such as Doppler gel are removed.

A plastic adhesive membrane or a thick layer of gauze can be applied around the leech(es) to prevent detached leeches from attaching themselves in other parts of the skin or even under the flap, to fall inside the dressing around the wound or other parts of the patient's body or on the bed, which could upset the patient and be unpleasant for other patients nearby.

In general leeches should be applied on the darker spots of the reattached body parts or flaps. In the case of reimplanted fingers, leeches could be also placed on the region of the removed nail.

**Figure 1:**
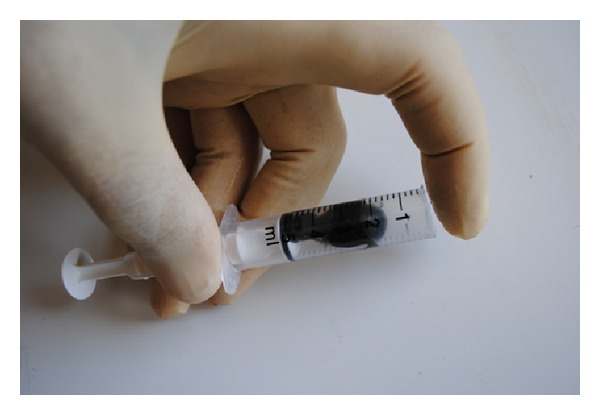
Application of a leech to the area to be treated using a syringe.

The leeches are usually placed on a given spot of the skin using a 5 mL syringe. For this purpose, the nozzle of the syringe is removed using a scissor or scalpel. The leech is placed in the barrel of the syringe and the open end of the syringe is placed on the area to be treated. When the leech starts feeding, the syringe is removed gently (Figure 1).

**Figure 2:**
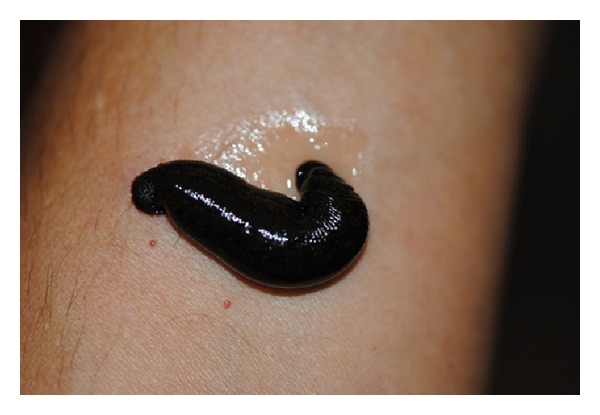
Feeding leech: transparent liquid can be seen oozing from the body of the leech.

Leeches normally start feeding immediately, although in some cases the skin has to be punctured with a sterile needle so that oozing blood will stimulate the leeches to feed. Pricking the site to be treated could also demonstrate whether there is sufficient blood-flow in the area. When the leech refuses to feed in a given place, the syringe is moved to the neighboring area, until an appropriate place is found as close as possible to the congested area. Depending on the intensity of blood-flow in the area, feeding can last for 30–90 min. During feeding drops of clear liquid can be seen oozing from the leech (Figure 2); this is the superfluous water in blood, which the leeches remove to concentrate the red blood cells in their digestive tract.

During hirudotherapy the patient should be under permanent surveillance by a healthcare provider; leeches may seek other places to suck blood or after feeding may drop into the surrounding area. After autodetachment, the leeches are killed in 70% ethyl alcohol and are disposed in bags for biological waste.

In cases of intraoral leeching, the path to the oropharynx should be blocked with gauze to prevent leech migration into the more distal aerodigestive tract, and the perioperative tracheotomy is left in position to protect the airway [29]. Specially shaped glass containers can be used for this purpose [33].

Attention should be paid to leeches, which attach but do not change in size and have no visible gut peristalsis within 30 min after their attachment. It could be assumed that they are only attached but not feeding and should be replaced with other leeches or they should be transferred to other parts of the treated area. The use of active (swimming) and larger leeches could be of help.

## 5. Treatment Procedure

Depending on the severity and the size of the congestion, 1–10 leeches are used for each treatment, although some authors recommend higher numbers of leeches. The degree of venous congestion is estimated from the percentage of violaceous color of flap skin pedicle, testing capillary refill, and color of the blood oozing from the bite site or after having been pierced with a needle. At the beginning of the treatment, the patient might need two or more treatments per day. Chepeha et al. [29] used a protocol according to which leech placement was continuous (3 leeches per hour) and tapered slowly according to clinical assessment of inosculation. In the Iowa Head and Neck Protocol [30], leeches are applied every 2 hours. The number of treatments per day depends also on the bleeding of previous bite sites. In cases where the bleeding stops shortly after the leeches detach, or when leeches do not become fully engorged, a more aggressive treatment should be followed by using a larger number of leeches and more treatments per day.

Leech therapy is used until venous capillary return is established across the wound border by angiogenesis. Usually the treatment with leeches lasts for 2–6 days. In fact, the decision regarding the duration of the leech treatment is empiric, based on subjective appreciation of the color of the skin, capillary refill, and the color of bleeding after pinprick [12, 29, 34–36].

Flap monitoring consists of listening to the Doppler signal and examining the flap every 1-2 hrs until the flap is stabilized and thereafter every 2–4 hrs [29].

Antithrombotic therapy could be administered with aspirin, heparin sodium, and/or dextran 40 in parallel with hirudotherapy, although it should be noted that the leech saliva also demonstrates antithrombotic activity.

The patients may lose 5–15 mL of blood per leech, per session. However, the wound may continue to ooze up to 24 hours after the leech is removed. Accordingly, hematologic evaluations should be performed every 4 hrs and consist of complete blood cell count, partial thromboplastin time, and serum chemistry studies. However, the number of hematocrit checks depends on the number of leeches used, frequency of sessions, and total duration of therapy. In many cases, the patients have to receive blood transfusions when the hemoglobin level is lower than 8 g/dL [29].

After detachment of the leech, the bite areas could be cleaned hourly with gauze soaked in isotonic sodium chloride solution or heparin solution (5,000 U/mL) to stimulate hemorrhage from leech bite sites [22, 33].

## 6. Assessment

**Figure 3:**
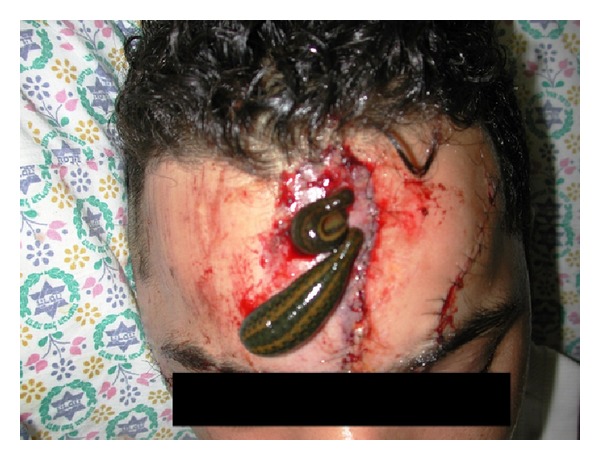
Treatment of a skin flap of the face, after removal of the skin with scars and coverage of the area with the enlarged, adjacent skin.

Successful salvage of decongested tissues with leeching has been reported in 70–80% of cases [10, 21, 37, 38]. In Israel, Mumcuoglu et al. [22] used hirudotherapy in 23 patients, 8–79 years old, presenting with venous congestion of revascularized or replanted fingers and free or local flaps. Of the 15 fingers, 10 fingers were saved (66.7%) (4 out of 9 replanted fingers and 6 out of 6 revascularized fingers), while 17 out of 18 flaps (94.4%) were salvaged (3 out of 4 free flaps and all 14 island and random flaps) (Figure 3). Similar results were reported from countries such as the USA [15, 22], UK [17], Germany [39], and S. Africa [31].

The degree of venous congestion can be estimated by describing the percentage of ruborous and violaceous color of the flap skin pedicle, testing capillary refill, and observing color and amount of blood oozing from leech bite sites. Serial photographs can help assess the intensity of venous congestion on a daily basis. The progress of treatment should be also documented.

It should be kept in mind that venous obstruction causes microcirculatory thrombosis, platelet trapping, and stasis. Thus, even after successful reanastomosis by leeches, secondary changes in the microcirculation can persist and prevent adequate outflow from being reestablished.

## 7. Side Effects and Contraindications

Modern leech therapy is generally recognized as a relatively safe and well-tolerated treatment modality. A patient who refuses to sign the consent form, refuses prophylactic treatment with antibiotics, or refuses blood transfusion should not be treated with leeches.

Although the bite of a leech is felt as a slight pain on the intact skin, this is not relevant in the case of recently reattached digits and flaps, where the skin is anesthetic.

**Figure 4:**
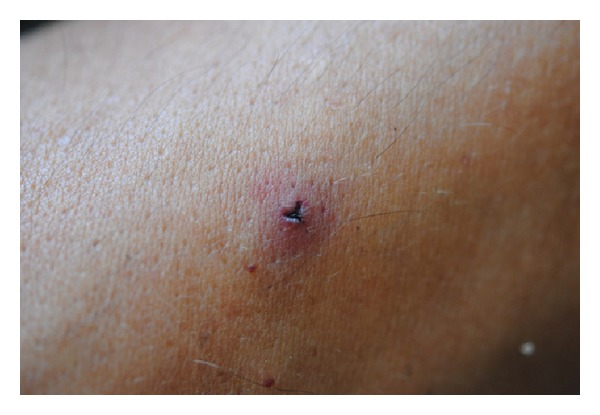
Y-shaped leech bite and the surrounding skin reaction.

Slight localized itching of the Y-shaped bite site (Figure 4) persisting for several hours and up to 3 days is the most common (37.3–75%) adverse effect of leech therapy. Use of 5% potassium permanganate, cold compresses, 10% baking soda paste, Golden Star balm, or Fenistil gel on the affected skin areas can be for more pronounced cases. In severe cases of generalized itching, topical corticosteroids and oral antihistamines should be prescribed.

Signs of regional lymphadenitis, slight swelling, and pain of regional lymph nodes on the side of leech application and subfebrile temperature can occur in 6.4–13.4% of the treated patients and usually appears after 3-4 leech applications. Apparently, such adverse reactions never appear when leeches are applied on oral, nasal, or vaginal mucosa. In very rare cases, allergic skin reactions have been observed. When leeches are applied to the esthetically important areas with thin skin and thin layers of subcutaneous tissue scarring after a leech bite could be a cosmetic problem. In some cases, application to a nearby mucosal surface could avoid these complications [33].

Symbiotic bacteria such as *Aeromonas hydrophila *and* Aeromonas veronii*, living in the intestinal tract of the leech, may cause infections in 4–20% of the patients, whose flaps or replanted digits are treated with leeches [40]. Leech-borne *Serratia marcescens *infections were also reported [24]. Accordingly, prophylactic treatment with antibiotics is necessary. *Aeromonas* infections can occur acutely (within 24 hours) or in a delayed fashion (up to 26 days) after the beginning of leech therapy. Clinical manifestations of *Aeromonas* infection vary from a minor wound infection to extensive tissue loss. It is important to stress that leech related *Aeromonas* infections more frequently develop in very sick and immunosuppressed patients. When applied on intact skin, for example, in patients treated for osteoarthritis, local pain, arterial hypertension, and different forms of spondylosis and dorsopathies, *Aeromonas* infections are extremely rare.

The excess bleeding after leeching can be of concern and transfusions may be needed, especially in patients with a tendency to hemorrhage, who suffer from anemia, or for those taking anticoagulants or platelet-inhibiting drugs. Blood transfusions are given based on a hemoglobin level of <8 g/dL. Usually, 3–6 units of packet blood cells are used to compensate for blood loss.

In very rare cases, thrombotic microangiopathy and renal failure have been reported when leeches were applied in patients with arterial insufficiency [33].

Isolated reports describing the appearance of syncopal status or orthostatic hypotension (mainly in older persons with initial sympathicotonia) at the start of or during leech therapy, have been reported. According to Michalsen et al. [41, 42] vasovagal attacks can occur in 0.1% of patients undergoing hirudotherapy, mainly in those with a history of developing such attacks or syncope during invasive procedures such as venopunction. It is highly recommended that patients drink plenty of fluids during hirudotherapy. In order to prevent those systemic side effects, hirudotherapy should be performed in a calm atmosphere, under constant blood pressure monitoring, and with the patient lying down.

Hirudotherapy can cause some negative psychological or emotional reactions in patients, especially when detached leeches fall inside the dressing or other parts of the patient's body. Less than 10% of patients undergoing leech therapy for osteoarthritis had initial qualms before treatment, which usually disappeared after the first treatment course. In fact many patients treated with leeches and who had successful treatment changed their attitude towards hirudotherapy in a positive way. Nevertheless, it might be necessary to prepare the patients psychologically before the application of leeches [41, 42].

Contraindications related to hirudotherapy include arterial insufficiency, hemophilia, hemorrhagic diathesis, hematological malignancies, expressed and firm anemia, expressed and firm hypotension, sepsis, HIV-infection, decompensated forms of hepatobiliary diseases, any form of cachexia, and individual intolerance to leeches. Leech therapy is also not recommended in pregnancy and lactation, in patients with an unstable medical status, history of allergy to leeches or severe allergic diathesis, disposition to keloid scar formation, arterial insufficiency, and in those using anticoagulants, immunosuppressants, and some vasoactive drugs such as *Ginkgo biloba* products [33].

## 8. Mechanisms Involved 

Hirudotherapy depends on the following main properties of medicinal leeches: the blood-letting action during active suction of blood, passive oozing of the wound, and injection of biologically active substances with the saliva into the host.

The saliva of *H. medicinalis* contains more than 100 bioactive substances, including coagulation inhibitors, platelet aggregation inhibitors, vasodilators, and anaesthetizing, antimicrobial and anti-inflammatory agents [43, 44].

One of the most important ingredients is hirudin, which is the principal anticoagulant responsible for enhanced bleeding and prevention of coagulation. In addition to hirudin, leeches secrete two inhibitors of Factor Xa responsible for the conversion of prothrombin to thrombin [45]. Furthermore, leech saliva is an effective platelet aggregation inhibitor due to the presence of active ingredients such as calin, apyrase, platelet activating factor (PAF-) antagonist, collagenase, and prostaglandin. Their main function is preventing the ingested blood from congealing within the leech's gut. The medical benefit to the patient is a sustained local bleeding that can last several hours after the end of each leech session.

The saliva of the medicinal leech also contains proteinase inhibitors, such as bdellins [46], eglin, inhibitors of *α*-chymotrypsin, subtilisin, and the granulocytic neutral proteases-elastase and cathepsin G [47, 48], responsible for the anti-inflammatory effect of leeching.

Medicinal leeches also secrete hirustasin, which selectively inhibits tissue kallikreins that are largely responsible for the maintenance of a normal level of blood pressure. Hirustasin can also play a role in the intrinsic coagulation process [49].

The anti-inflammatory and analgesic properties of leeches are subjects of modern hirudobiochemistry and hirudopharmacology and in many aspects are associated with the blockage of amidolytic and kininogenase activities of plasma kallikrein, resulting in prevention of pain or pain relief during leech sessions [50].

Leeches may also secrete a vasodilative, histamine-like substance, which increases the inflow of blood after a leech bite and reduces local swelling [45].

Hyaluronidase, which is known as the “spreading factor,” can degrade tissue hyaluronic acid, thus facilitating the infiltration and diffusion of the remaining ingredients of leech saliva into the congested tissue. Tissue permeability, restored with the help of hyaluronidase, promotes the elimination of tissue- and circulatory-hypoxia as well as local swelling [51].

The persistent bleeding largely potentiates tissue decongestion and leads to loss of blood, relief of capillary net, decrease in venous congestion, decompression of the nerve trunks and endings, increase in lymph flow, positive changes of local hemodynamics, amelioration of hemorheology, increase of oxygen supply, improvement of tissue metabolism, and elimination of tissue ischemia [33].

## 9. Conclusions

In summary, hirudotherapy is a safe, easy to use, beneficial, and cost-effective treatment modality to save reattached body parts and flaps in reconstructive plastic surgery. The early recognition of flap failure and initiation of leech therapy is of paramount importance. Prophylactic treatment with antibiotics and continuous monitoring of blood parameters are necessary.

## References

[1] Deganc M, Zdravic F (1960). Venous congestion of flaps treated by application of leeches. *British Journal of Plastic Surgery*.

[2] Michalsen A, Roth M, Dobos G (2007). *Medicinal Leech Therapy*.

[3] Grassberger M, Sherman RA, Gileva O, Kim CMH, Mumcuoglu KY (2013). *Biotherapy—History, Principles and Practice: A Practical Guide to the Diagnosis and Treatment of Disease Using Living Organisms*.

[4] Hartrampf CR, Drazan L, Noel RT (1993). A mechanical leech for transverse rectus abdominis musculocutaneous flaps. *Annals of Plastic Surgery*.

[5] Frodel JL, Barth P, Wagner J (2004). Salvage of partial facial soft tissue avulsions with medicinal leeches. *Otolaryngology*.

[6] Lim CL (1986). Successful transfer of ’free’ microvascular superficial temporal artery flap with no obvious venous drainage and use of leeches for reducing venous congestion: case report. *Microsurgery*.

[7] Baudet J (1991). The use of leeches in distal digital replantation. *Blood Coagulation & Fibrinolysis*.

[8] Soucacos PN, Beris AE, Malizos KN, Kabani CT, Pakos S (1994). The use of medicinal leeches, *Hirudo medicinalis*, to restore venous circulation in trauma and reconstructive microsurgery. *International Angiology*.

[9] de Chalain T, Cohen SR, Burstein FD (1995). Successful use of leeches in the treatment of purpura fulminans. *Annals of Plastic Surgery*.

[10] de Chalain TMB (1996). Exploring the use of the medicinal leech: a clinical risk-benefit analysis. *Journal of Reconstructive Microsurgery*.

[11] Utley DS, Koch RJ, Goode RL (1998). The failing flap in facial plastic and reconstructive surgery: role of the medicinal leech. *Laryngoscope*.

[12] Weinfeld AB, Kattash M, Grifka R, Friedman JD (1998). Leech therapy in the management of acute venous congestion of an infant’s lower limb. *Plastic and Reconstructive Surgery*.

[13] Trovato MJ, Agarwal JP (2008). Successful replantation of the ear as a venous flap. *Annals of Plastic Surgery*.

[14] Ausen K, Pavlovic I (2011). Flaps pedicled on the superficial temporal artery and vein in facial reconstruction: a versatile option with a venous pitfall. *Journal of Plastic Surgery and Hand Surgery*.

[15] Koch M (2011). Comment on F. Riede , W. Koenen, S. Goerdt, H. Ehmke, J. Faulhaber “Medicinal leeches for the treatment of venous congestion and hematoma after plastic reconstructive surgery” in Journal der Deutschen Dermatologischen Gesellschaft 2010; 11: 881–889. *Journal der Deutschen Dermatologischen Gesellschaft*.

[16] Nguyen MQ, Crosby MA, Skoracki RJ, Hanasono MM (2012). Outcomes of flap salvage with medicinal leech therapy. *Microsurgery*.

[17] Whitaker IS, Oboumarzouk O, Rozen WM (2012). The efficacy of medicinal leeches in plastic and reconstructive surgery: a systematic review of 277 reported clinical cases. *Microsurgery*.

[18] Senchenkov A, Jacobson SR (2013). Microvascular salvage of a thrombosed total ear replant. *Mirosurgery*.

[19] Chen YC, Chan FC, Hsu CC, Lin YT, Chen CT, Lin CH (2013). Fingertip replantation without venous anastomosis. *Annals of Plastic Surgery*.

[20] Vural E, Key JM (2001). Complications, salvage, and enhancement of local flaps in facial reconstruction. *Otolaryngologic Clinics of North America*.

[21] Whitaker IS, Izadi D, Oliver DW, Monteath G, Butler PE (2004). *Hirudo medicinalis* and the plastic surgeon. *British Journal of Plastic Surgery*.

[22] Mumcuoglu KY, Pidhorz C, Cohen R, Ofek A, Lipton HA (2007). The use of the medicinal leech, *Hirudo medicinalis*, in the reconstructive plastic surgery. *The Internet Journal of Plastic Surgery*.

[23] Mumcuoglu KY, Huberman L, Cohen R (2010). Elimination of symbiotic *Aeromonas* spp. from the intestinal tract of the medicinal leech, *Hirudo medicinalis*, using ciprofloxacin feeding. *Clinical Microbiology and Infection*.

[24] Pereira JA, Greig JR, Liddy H, Ion L, Moss ALH (1998). Leech-borne Serratia marcescens infection following complex hand injury. *British Journal of Plastic Surgery*.

[25] Ardehali B, Hand K, Nduka C, Holmes A, Wood S (2006). Delayed leech-borne infection with *Aeromonas hydrophilia* in escharotic flap wound. *Journal of Plastic, Reconstructive and Aesthetic Surgery*.

[26] Whitaker IS, Kamya C, Azzopardi EA, Graf J, Kon M, Lineaweaver WC (2009). Preventing infective complications following leech therapy: is practice keeping pace with current research?. *Microsurgery*.

[27] Schnabl SM (2011). Comment on F. Riede, W. Koenen, S. Goerdt, H. Ehmke,J. Faulhaber “Medicinal leeches for the treatment of venous congestion and hematoma after plastic reconstructive surgery” in Journal der Deutschen Dermatologischen Gesellschaft 2010; 11: 881–889. *Journal der Deutschen Dermatologischen Gesellschaft*.

[28] Wilmer A, Slater K, Yip J, Carr N, Grant J (2013). The role of leech water sampling in choice of prophylactic antibiotics in medical leech therapy. *Microsurgery*.

[29] Chepeha DB, Nussenbaum B, Bradford CR, Teknos TN (2002). Leech therapy for patients with surgically unsalvageable venous obstruction after revascularized free tissue transfer. *Archives of Otolaryngology*.

[30] Colombo MR https://wiki.uiowa.edu/display/protocols/Leech+Therapy+-+Anticoagulation+Protocols.

[31] van Wingerden JJ, Oosthuizen JH (1997). Use of the local leech Hirudo michaelseni in reconstructive plastic and hand surgery. *South African Journal of Surgery*.

[32] Siddall ME, Min G-S, Fontanella FM, Phillips AJ, Watson SC (2011). Bacterial symbiont and salivary peptide evolution in the context of leech phylogeny. *Parasitology*.

[33] Gileva OS, Mumcuoglu KY, Grassberger M, Sherman RA, Gileva O, Kim CMH, Mumcuoglu KY (2013). Hirudotherapy. *Biotherapy—History, Principles and Practice: A Practical Guide to the Diagnosis and Treatment of Disease Using Living Organisms*.

[34] Brody GA, Maloney WJ, Hentz VR (1989). Digit replantation applying the leech *Hirudo medicinalis*. *Clinical Orthopaedics and Related Research*.

[35] Henderson HP, Matti B, Laing AG (1983). Avulsion of the scalp treated by microvascular repair: the use of leeches for post-operative decongestion. *British Journal of Plastic Surgery*.

[36] Mutimer KL, Banis JC, Upton J (1987). Microsurgical reattachment of totally amputated ears. *Plastic and Reconstructive Surgery*.

[37] Durrant C, Townley WA, Ramkumar S, Khoo CTK (2006). Forgotten digital tourniquet: salvage of an ischaemic finger by application of medicinal leeches. *Annals of the Royal College of Surgeons of England*.

[38] Hayden RE, Phillips JG, McLear PW (1988). Leeches. Objective monitoring of altered perfusion in congested flaps. *Archives of Otolaryngology*.

[39] Gröbe A, Michalsen A, Hanken H, Schmelzle R, Heiland M, Blessmann M (2012). Leech therapy in reconstructive maxillofacial surgery. *Journal of Oral and Maxillofacial Surgery*.

[40] Lineaweaver WC, Furnas H, Follansbee S (1992). Postprandial *Aeromonas hydrophila* cultures and antibiotic levels of enteric aspirates from medicinal leeches applied to patients receiving antibiotics. *Annals of Plastic Surgery*.

[41] Michalsen A, Deuse U, Esch T, Dobos G, Moebus S (2001). Effect of leeches therapy (*Hirudo medicinalis*) in painful osteoarthritis of the knee: a pilot study. *Annals of the Rheumatic Diseases*.

[42] Michalsen A, Klotz S, Lüdtke R, Moebus S, Spahn G, Dobos GJ (2003). Effectiveness of leech therapy in osteoarthritis of the knee: a randomized, controlled trial. *Annals of Internal Medicine*.

[43] Eldor A, Orevi M, Rigbi M (1996). The role of the leech in medical therapeutics. *Blood Reviews*.

[44] Siddall ME, Trontelj P, Utevsky SY, Nkamany M, Macdonald KS (2007). Diverse molecular data demonstrate that commercially available medicinal leeches are not *Hirudo medicinalis*. *Proceedings of the Royal Society B*.

[45] Rigbi M, Orevi M, Eldor A (1996). Platelet aggregation and coagulation inhibitors in leech saliva and their roles in leech therapy. *Seminars in Thrombosis and Hemostasis*.

[46] Fritz H, Oppitz KH, Gebhardt M, Oppitz I, Werle E, Marx R (1969). On the presence of a trypsin-plasmin inhibitor in hirudin. *Hoppe-Seyler’s Zeitschrift fur Physiologische Chemie*.

[47] Seemueller U, Meier M, Ohlsson K, Müller HP, Fritz H (1977). Isolation and characterisation of a low molecular weight inhibitor (of chymotrypsin and human granulocytic elastase and cathepsin G) from leeches. *Hoppe-Seyler’s Zeitschrift fur Physiologische Chemie*.

[48] Seemuller U, Dodt J, Fink E, Barettand AJ, Salvesen G (1986). Proteinase inhibitors of the leech *Hirudo medicinalis* (hirudins, bdellins, eglins). *Proteinase Inhibitors*.

[49] Sollner C, Mentele R, Eckerskorn C, Fritz H, Sommerhoff CP (1994). Isolation and characterization of hirustasin, an antistasin-type serine-proteinase inhibitor from the medical leech Hirudo medicinalis. *European Journal of Biochemistry*.

[50] Baskova IP, Khalil S, Nartikova VF, Paskhina TS (1992). Inhibition of plasma kallikrein, kininase and kinin-like activities of preparations from the medicinal leeches. *Thrombosis Research*.

[51] Hovingh P, Linker A (1999). Hyaluronidase activity in leeches (Hirudinea). *Comparative Biochemistry and Physiology B*.

